# Influenza vaccination and secondary prevention of cardiovascular disease among Taiwanese elders—A propensity score-matched follow-up study

**DOI:** 10.1371/journal.pone.0219172

**Published:** 2019-07-01

**Authors:** Hao-Hsin Wu, Yea-Yuan Chang, Shu-Chen Kuo, Yung-Tai Chen

**Affiliations:** 1 Division of Infection Control and Biosafety, Centers for Disease Control, Taipei, Taiwan; 2 Division of Infectious Diseases, Department of Internal Medicine, National Yang-Ming University Hospital, Yilan County, Taiwan; 3 National Institute of Infectious Diseases and Vaccinology, National Health Research Institutes, Miaoli County, Taiwan; 4 Divisions of Nephrology, Taipei Veterans General Hospital, Taipei, Taiwan; 5 Department of Medicine, Taipei City Hospital Heping Fuyou Branch, Taipei, Taiwan; Örebro University Hospital, SWEDEN

## Abstract

The present study aimed to evaluate the association between influenza vaccination and the secondary prevention of cardiovascular disease (CVD) among elderly persons. This retrospective cohort study used the Geriatric Dataset of Taiwan’s National Health Insurance Research Database (2000–2013). Patients aged ≥ 65 years who had been hospitalized for the first episodes of myocardial infarction were eligible. The vaccinated cohort comprised patients who received one dose of influenza vaccine within 180 days after discharge. The unvaccinated cohort included those who did not receive influenza vaccination and was propensity score–matched (1:1) for known CVD risk factors. All-cause death, acute myocardial infarction or cardiovascular death, and hospitalization for heart failure were assessed 1 year after the 181^st^ day after hospital discharge. Compared with the matched cohort (*n* = 4,350), the vaccinated cohort (*n* = 4,350) had significantly lower incidences of all-cause death (hazard ratios [HR] 0.82, 95% CI [confidence interval] 0.73–0.92), myocardial infarction or cardiovascular death (HR 0.84, 95% CI 0.74–0.96), and hospitalization for heart failure (HR 0.83, 95% CI 0.74–0.92). The association between influenza vaccination and reduction of CVDs was similar across different subgroups. Cumulative incidence curves of the CVDs of interest for the two cohorts separated within the initial 3 months of follow-up (*P* < 0.05). Influenza vaccination was associated with a reduced risk of CVD in the elderly population with previous myocardial infarction.

## Introduction

Cardiovascular disease (CVD) is the leading cause of mortality worldwide, including in Taiwan, where it accounts for approximately 11% of all deaths [1]. Patients with previous myocardial infarction (MI) are at the greatest risk of recurrent CVD and have six times the annual death rate compared with people without previous MI [2]. Given the high disease burden of CVD, secondary prevention through the identification and mitigation of risk factors is a priority. Several treatment modalities targeting traditional risk factors, including prescription of anti-hypertensive drugs or lipid-lowering medications, and control of blood sugars, have been demonstrated to prevent CVD recurrence [3, 4].

Interest remains in atypical cardiovascular risk factors, especially the potential association between influenza and subsequent CVD [5]. This association may be attributed to altered endothelial function incurred by influenza-related inflammatory and procoagulant stimulus, and vaccination theoretically mitigates the risk [5–8]. Several randomized clinical trials [9–11] and meta-analyses [12, 13] have demonstrated the benefits of influenza vaccination in the secondary prevention of overall mortality, cardiovascular events, cardiovascular death, and hospitalization. Although 60% of recurrent CVD cases are in elderly patients [14], few studies have examined whether influenza vaccination in the elderly population is beneficial with respect to CVD [9–11]. Two randomized clinical trials enrolled patients with a mean age of around 65 years, but these populations were heterogeneous in age [9, 11]. In one study, subgroup analysis showed that patients aged > 65 years benefited from influenza vaccination in terms of the secondary prevention of CVDs, but the number of cases was limited [11]. Results from the general population should not be extrapolated to the elderly population, as the protective effect of influenza vaccination has been shown to decrease with age [15].

Therefore, we performed a population-based, propensity score–matched cohort study to assess the association between influenza vaccination and the secondary prevention of cardiovascular disease (CVD) among elderly persons. Taiwan’s National Health Insurance Research Database (NHIRD, 2000–2013) was used to identify matched vaccinated and unvaccinated cohorts with histories of MI during a 14-year period.

## Materials and methods

### Data source

Taiwan’s National Health Insurance covers 99.6% of the population because of mandatory universal enrollment. All diagnoses [in accord with the *International Classification of Diseases*, *Ninth Revision*, *Clinical Modification* (ICD-9-CM)], procedures, and medications for patients enrolled in the insurance system are recorded and stored in the NHIRD, maintained by the National Health Research Institutes. The National Health Insurance Act prohibits the retrieval of data on all patients aged ≥ 65 years. The Geriatric Dataset contains complete data for a random sample of beneficiaries aged ≥ 65 years (sampling ratio 1:3) extracted from the original NHIRD. The study was approved and exempted from the informed consent requirement by the institutional review board of the National Health Research Institute (EC1030804-E) because the data set comprised deidentified secondary data.

### Study population and design

Taiwanese patients aged ≥ 65 years who were admitted to hospitals with diagnoses of acute MI (ICD-9-CM 410.X) during 2000–2013 were eligible for enrollment. Data for each eligible individual were further extracted from Geriatric Dataset during 1995 to 1999 to ensure that all individuals were available for a 5-year follow-up before enrollment. Only patients who experienced the first episodes of MI were included. The index date was the 181^st^ day after hospital discharge. The vaccinated cohort comprised patients who received one dose of intramuscular influenza vaccine within the period between the date of discharge and the index date, and the unvaccinated cohort comprised those who did not receive the influenza vaccine during the designated period. To control for potential confounding due to imbalance in clinical characteristics between the vaccinated and unvaccinated cohorts, we used propensity score matching [16] to assemble comparable cohorts. A logistic regression model including age, sex, socioeconomic status, pre-existing comorbidities, and concomitant medications as covariates was used to predict propensity scores. A propensity score [16, 17] was then used to identify a unique matched counterpart from the original unvaccinated cohort for each vaccinated patient (1:1 matching). Both cohorts were followed for 12 months after the index date or until loss to follow-up. The endpoints were all-cause death, hospitalization for cardiac failure (ICD-9-CM 428.XX), and a composite outcome consisting of MI (ICD-9-CM 410.XX) and cardiovascular death. To further evaluate the temporal relationship between influenza vaccination and outcome events, we performed sensitivity analyses for two timeframes: 12 months after vaccination and within the same influenza seasons. Hospitalization for upper gastrointestinal bleeding was used as a negative control event [18].

### Definition of covariates

Pre-existing comorbidities associated with CVD or the prognoses of patients with CVD [4, 19–22] were defined according to the ICD-9-CM, and included hypertension, heart failure, diabetes, peptic ulcer disease, liver disease, chronic kidney disease, atrial fibrillation, cerebrovascular disease, dyslipidemia, valvular heart disease, cancer, autoimmune disease, and substance abuse. We also collected information on concomitant use of medications associated with CVD or the above-mentioned comorbidities, including antiplatelet agents (including aspirin, clopidogrel, and ticlopidine), angiotensin-converting enzyme inhibitors/angiotensin II receptor blockers, β blockers, calcium channel blockers, diuretics, insulin, oral anti-hyperglycemic agents, statins, and steroids [4, 22]. Previous studies have suggested that the prognosis of CVD is affected by the Charlson comorbidity score [21, 23] and socioeconomic status [24], and that the frequency of outpatient department use is associated with receipt of the influenza vaccination [25]. Therefore, these factors were also included in the logistic regression model for propensity score prediction.

### Statistical analysis

We used the standardized difference (StD) to compare the means of continuous and binary variables between the vaccinated and unvaccinated cohorts [17]. Imbalance in the characteristics of the two cohorts was defined as an absolute StD > 0.04. The incidence rates of outcome events were calculated using Poisson distribution. Cumulative incidence curves were assessed using the Kaplan–Meier method and compared using the log-rank test. A Cox proportional hazards model was used to calculate hazard ratios (HRs) and 95% confidence intervals (CIs) to evaluate associations between outcomes and vaccination. Competing-risks regression using Fine and Gray’s model was also performed [26]. We assessed the terms of interaction between influenza vaccination and the variables to determine whether the effect of vaccination differed between groups. Two-sided tests were used to explore statistical significance, at the level of *P* < 0.05. For interaction terms, Bonferroni-corrected *P* values < 0.005 (0.05/10) were considered to be significant. The Microsoft SQL Server 2008 R2 (Microsoft) was used for data linkage, processing, and sampling. All analyses were performed using SAS software version 9.2 (SAS Institute, North Carolina, USA).

## Results

During the study period, a total of 29,066 patients aged ≥ 65 years with first episodes of acute MI was eligible for inclusion. Among the 4,554 vaccinated patients, 204 patients could not be well matched according to propensity scores. Therefore, we enrolled 4,350 vaccinated patients and 4,350 unvaccinated patients. The mean age of the vaccinated cohort was 76.3 (standard deviation 6.5) years. The majority of these patients was male (64.9%) and had high Charlson Comorbidity Index scores. The characteristics of both cohorts are detailed in Table 1. The matched cohorts were well balanced in terms of all observed covariates.

**Table 1:** Baseline characteristics of elderly participants with histories of myocardial infarction.

Characteristic	Before Propensity Score Matching			Propensity Score–Matched		
	Vaccination (*n* = 4,554)	No Vaccination (*n* = 24,512)	StD^a^	Vaccination (*n* = 4,350)	No Vaccination (*n* = 4,350)	StD^a^
Mean age (years)	76.3 (6.4)	76.0 (7.0)	0.05	76.3 (6.5)	76.2 (6.5)	0.01
Male	2,998 (65.83)	15,190 (61.95)	0.08	2,823 (64.90)	2,845 (65.40)	–0.01
Month of myocardial infarction						
January	725 (15.92)	1,392 (5.68)	0.34	677 (15.56)	684 (15.72)	<–0.01
February	693 (15.22)	1,268 (5.17)	0.34	636 (14.62)	656 (15.08)	–0.01
March	657 (14.43)	1,472 (6.01)	0.28	642 (14.76)	658 (15.13)	–0.01
April	285 (6.26)	1,752 (7.15)	–0.04	285 (6.55)	298 (6.85)	–0.01
May	84 (1.84)	2,254 (9.20)	–0.33	84 (1.93)	89 (2.05)	–0.01
June	26 (0.57)	2,449 (9.99)	–0.43	26 (0.60)	26 (0.60)	<0.01
July	8 (0.18)	2,969 (12.11)	–0.51	8 (0.18)	8 (0.18)	<0.01
August	0 (0.00)	2,914 (11.89)	–0.52	0 (0.00)	0 (0.00)	<0.01
September	7 (0.15)	2,780 (11.34)	–0.50	7 (0.16)	9 (1.21)	–0.01
October	525 (11.53)	2,158 (8.80)	0.09	524 (12.05)	478 (10.99)	0.03
November	788 (17.30)	1,577 (6.43)	0.34	745 (17.13)	751 (17.26)	<–0.01
December	756 (16.60)	1,527 (6.23)	0.33	716 (16.46)	693 (15.93)	0.01
Monthly income (NT dollars)						
Dependent	1,627 (35.73)	9,971 (40.66)	–0.10	1,597 (36.71)	1,595 (36.67)	<0.01
<19,100	1,404 (30.83)	6,583 (26.85)	0.09	1,304 (29.98)	1,314 (30.21)	–0.01
19,100–41,999	1,495 (32.83)	7,762 (31.62)	0.03	1,422 (32.69)	1,410 (32.41)	0.01
≥42,000	28 (0.61)	196 (0.80)	–0.02	27 (0.62)	31 (0.71)	–0.01
Urbanization level^b^						
1	1,316 (28.90)	6,946 (28.33)	0.01	1,265 (29.08)	1,271 (29.22)	<–0.01
2	2,926 (64.25)	16,072 (65.54)	–0.03	2,803 (64.44)	2,794 (64.23)	<0.01
3	264 (5.80)	1,210 (4.93)	0.04	235 (5.40)	239 (5.49)	<–0.01
4 (rural)	48 (1.05)	284 (1.16)	–0.01	47 (1.08)	46 (1.06)	<0.01
Outpatient visits in the past year						
0–5	12 (0.26)	1,302 (5.31)	–0.31	12 (0.28)	16 (0.37)	–0.02
6–10	99 (2.17)	1,509 (6.16)	–0.20	99 (2.28)	104 (2.39)	–0.01
11–15	269 (5.91)	2,606 (10.63)	–0.17	269 (6.18)	284 (6.53)	–0.01
16–20	490 (10.76)	3,140 (12.81)	–0.06	489 (11.24)	461 (10.60)	0.02
>20	3,684 (80.90)	15,955 (65.09)	0.36	3,481 (80.02)	3,485 (80.11)	<–0.01
Charlson Comorbidity Index score^c^	6.1 (3.1)	5.8 (3.2)	0.10	6.1 (3.1)	6.1 (3.1)	0.01
Concomitant medications						
Antiplatelet agent	3,548 (77.91)	17,950 (73.20)	0.11	3,365 (77.36)	3,351 (77.03)	0.01
ACEI/ARB	2,501 (54.92)	12,495 (50.96)	0.08	2,383 (54.78)	2,376 (54.62)	<0.01
β blocker	1,944 (42.69)	10,287 (41.95)	0.02	1,862 (42.80)	1,864 (42.85)	<–0.01
Calcium channel blocker	1,652 (36.28)	7,923 (32.31)	0.08	1,551 (35.66)	1,573 (36.16)	–0.01
Diuretic	1,603 (35.20)	8,699 (35.48)	<–0.01	1,540 (35.40)	1,552 (36.58)	–0.01
Insulin	349 (7.66)	1,873 (7.64)	0.01	334 (7.68)	347 (7.98)	–0.01
Oral anti-hyperglycemic drug	1,219 (26.77)	6,358 (25.93)	0.02	1,161 (26.69)	1,129 (35.95)	0.02
Statin	1,296 (28.46)	6,956 (28.37)	<0.01	1,248 (38.69)	1,242 (28.55)	<0.01
Steroid	694 (15.24)	3,241 (13.22)	0.06	656 (15.08)	665 (15.29)	<–0.01
Comorbidities						
Hypertension	4,111 (90.27)	21,127 (86.16)	0.13	3,918 (90.07)	3,909 (89.86)	0.01
Heart failure	2,131 (46.79)	12,025 (49.04)	–0.05	2,062 (47.40)	2,068 (47.54)	<–0.01
Diabetes mellitus	2,443 (53.65)	13,003 (53.03)	0.01	2,334 (53.66)	2,327 (53.49)	<0.01
Peptic ulcer disease	2,788 (61.22)	13,579 (55.38)	0.12	2,647 (60.85)	2,642 (60.74)	<0.01
Liver disease	1,312 (28.81)	5,935 (24.20)	0.10	1,236 (28.41)	1,224 (28.14)	0.01
Chronic kidney disease	1,515 (33.07)	7,740 (31.56)	0.04	1,442 (33.15)	1,473 (33.86)	–0.02
Atrial fibrillation	678 (14.89)	3,690 (15.05)	–0.01	660 (15.17)	673 (15.47)	–0.01
Cerebrovascular disease^d^	2,293 (50.35)	11,313 (46.14)	0.08	2,168 (49.84)	2,164 (49.75)	<0.01
Dyslipidemia	2,627 (57.69)	13,367 (54.51)	0.06	2,505 (57.59)	2,464 (56.64)	0.02
Valvular heart disease	1,036 (22.75)	5,371 (21.90)	0.02	991 (22.78)	1,014 (23.31)	–0.01
Cancer	752 (16.51)	3,683 (15.02)	0.04	718 (16.51)	703 (16.16)	0.01
Autoimmune disease	195 (1.28)	982 (4.00)	0.01	188 (4.32)	181 (4.16)	0.01
Drug abuse	87 (1.91)	501 (2.04)	–0.01	85 (1.95)	85 (1.95)	<0.01
Propensity score	0.36 (0.14)	0.12 (0.16)	1.57	0.35 (0.14)	0.35 (0.14)	<0.01

Data are expressed as number (%) or mean (standard deviation).

StD, standardized difference; ACEI, angiotensin-converting-enzyme inhibitor; ARB, angiotensin II receptor blocker.

^a^ Imbalance defined as absolute value > 0.04.

^b^ Defined by the Taiwan National Health Research Institute, with level 1 designating the most urbanized areas and level 4 designating the least urbanized areas.

^c^ Used to determine overall systemic health, with each increment of increase associated with a stepwise increase in cumulative mortality.

^d^ Defined as ICD-9-CM codes 433.X, 434.X, 436, 431, and 432.

**Fig 1:**
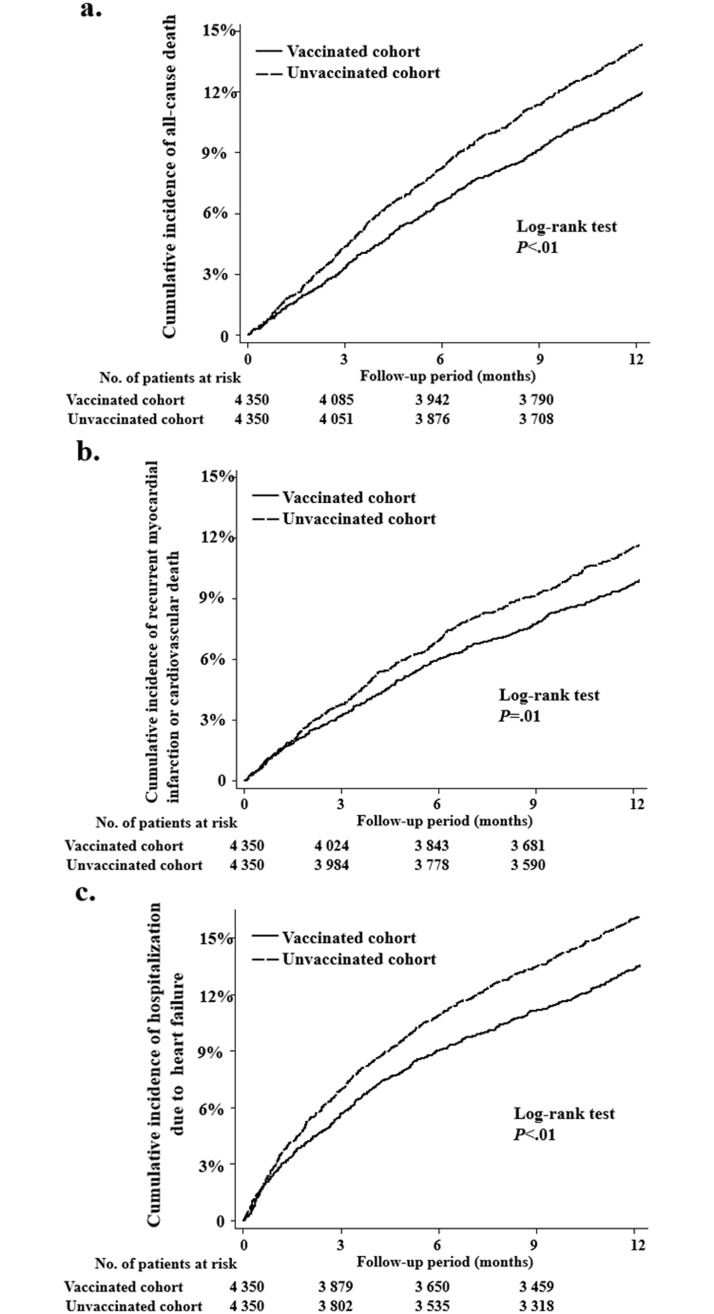
Cumulative incidences of cardiovascular outcomes among elderly participants with histories of myocardial infarction. (a) All-cause death. (b) Recurrent myocardial infarction or cardiovascular death. (c) Hospitalization due to heart failure.

The numbers and incidence rates of outcome events for the two propensity score–matched cohorts are presented in Table 2. About 11.6% of the vaccinated cohort and 13.9% of unvaccinated cohort died during the 1-year follow-up period. Compared with the unvaccinated cohort, the vaccinated cohort had significantly lower rates of all-cause mortality (HR 0.82, 95% CI 0.73–0.92), MI or cardiovascular death (HR 0.84, 95% CI 0.74–0.96), and hospitalization for cardiac failure (HR 0.83, 95% CI 0.74–0.92). In competing-risk survival analysis, the incidence rates of MI or cardiovascular death (HR 0.85, 95% CI 0.75–0.97) and hospitalization for cardiac failure (HR 0.84, 95% CI 0.75–0.94) were also significantly lower in the vaccinated cohort. The incidence rate of hospitalization for upper gastrointestinal bleeding did not differ significantly between groups in either model. Kaplan–Meier curves of cumulative incidence are shown in Fig 1. For all three endpoints, the curves of the two groups separated gradually during the first 3 months of the follow-up period and differed significantly at the end of follow-up. The effects of vaccination on all outcome events in patient subgroups are shown in Tables 3–5. The benefit of influenza vaccination in terms of all-cause death, recurrent MI or cardiovascular death, and hospitalization due to heart failure were consistent across different subgroups. The incidences of relevant cardiovascular events remained lower in the vaccinated cohort in both sensitivity analyses (S1 and S2 Tables).

**Table 2:** Incidence and risk of outcomes among elderly participants with histories of myocardial infarction.

	Vaccinated cohort (*n* = 4,350)			Unvaccinated cohort (*n* = 4,350; Reference)			Crude		Competing risk	
Outcome	No. of events	Person-years	Incidence rate^a^	No. of events	Person-years	Incidence rate ^a^	Hazard ratio (95% CI)	p-value	Hazard ratio (95% CI)	p-value
All-cause death	504	3,936	12.81	606	3,877	15.63	0.82 (0.73–0.92)	<0.01		
Myocardial infarction or cardiovascular death	407	3,852	10.57	475	3,784	12.55	0.84 (0.74–0.96)	0.01	0.85 (0.75–0.97)	0.02
Hospitalization for heart failure	552	3,676	15.02	653	3,567	18.31	0.83 (0.74–0.92)	<0.01	0.84 (0.75–0.94)	<0.01
Hospitalization for upper gastrointestinal bleeding	68	3,903	1.74	69	3,848	1.79	0.97 (0.70–1.35)	0.88	0.99 (0.71–1.38)	0.94

CI, confidence interval.

^a^ per 10^2^ person-years.

**Table 3:** Risk of all-cause death among elderly participants with histories of myocardial infarction.

Characteristic	Odds ratio^a^ (95% CI)	p-value	Interaction p-value
Sex			
Male	0.83 (0.71–0.96)	0.01	0.90
Female	0.81 (0.67–0.98)	0.03	
Age, years			
65–75	0.81 (0.65–1.02)	0.07	0.97
≥75	0.82 (0.71–0.94)	0.01	
Hypertension			
Yes	0.80 (0.71–0.91)	<0.01	0.16
No	1.11 (0.72–1.71)	0.64	
Diabetes mellitus			
Yes	0.77 (0.66–0.90)	<0.01	0.21
No	0.90 (0.74–1.09)	0.28	
Dyslipidemia			
Yes	0.85 (0.72–1.00)	0.06	0.56
No	0.79 (0.67–0.94)	0.01	
Chronic kidney disease			
Yes	0.80 (0.67–0.95)	0.01	0.61
No	0.85 (0.72–0.99)	0.05	
ACEI/ARB use			
Yes	0.81 (0.68–0.98)	0.03	0.96
No	0.82 (0.71–0.96)	0.01	
Antiplatelet agent use^b^			
Yes	0.80 (0.69–0.93)	<0.01	0.65
No	0.85 (0.71–1.03)	0.09	
β blocker use			
Yes	0.72 (0.57–0.92)	0.01	0.25
No	0.85 (0.74–0.98)	0.02	
Statin use			
Yes	0.80 (0.66–1.24)	0.54	0.49
No	0.80 (0.71–0.91)	<0.01	
History of influenza vaccination administration			
Yes	0.76 (0.66–0.88)	<0.01	0.42
No	0.85 (0.68–1.07)	0.16	

CI, confidence interval; ACEI, angiotensin-converting-enzyme inhibitor; ARB, angiotensin II receptor blocker.

^a^ Versus matched counterparts.

^b^ Including aspirin, clopidogrel, and ticlopidine.

**Table 4:** Risk of recurrent myocardial infarction or cardiovascular death among elderly participants with histories of myocardial infarction.

Characteristic	Odds ratio^a^ (95% CI)	p-value	Interaction p-value
Sex			
Male	0.86 (0.73–1.02)	0.08	0.64
Female	0.81 (0.65–1.01)	0.06	
Age, years			
65–75	0.93 (0.74–1.20)	0.57	0.29
≥75	0.80 (0.68–0.94)	0.01	
Hypertension			
Yes	0.85 (0.74–0.97)	0.02	0.61
No	0.73 (0.40–1.31)	0.29	
Diabetes mellitus			
Yes	0.81 (0.69–0.96)	0.02	0.52
No	0.89 (0.72–1.10)	0.29	
Dyslipidemia			
Yes	0.84 (0.70–1.01)	0.06	0.94
No	0.85 (0.70–41.03)	0.10	
Chronic kidney disease			
Yes	0.88 (0.73–1.07)	0.20	0.53
No	0.81 (0.67–0.98)	0.03	
ACEI/ARB use			
Yes	0.83 (0.69–0.99)	0.04	0.76
No	0.86 (0.71–1.04)	0.12	
Antiplatelet agent use^b^			
Yes	0.83 (0.71–0.98)	0.03	0.82
No	0.87 (0.68–1.11)	0.25	
β blocker use			
Yes	0.69 (0.56–0.86)	< 0.01	0.03
No	0.95 (0.80–1.12)	0.51	
Statin use			
Yes	0.79 (0.59–1.06)	0.12	0.66
No	0.86 (0.74–0.99)	0.04	
History of influenza vaccination administration			
Yes	0.82 (0.70–0.96)	0.01	0.81
No	0.79 (0.61–1.02)	0.068	

CI, confidence interval; ACEI, angiotensin-converting-enzyme inhibitor; ARB, angiotensin II receptor blocker.

^a^ Versus matched counterparts.

^b^ Including aspirin, clopidogrel, and ticlopidine.

**Table 5:** Risk of hospitalization due to heart failure among elderly participants with histories of myocardial infarction.

Characteristic	Odds ratio^a^ (95% CI)	p-value	Interaction p-value
Sex			
Male	0.85 (0.74–0.98)	0.03	0.47
Female	0.78 (0.65–0.94)	0.01	
Age, years			
65–75	0.76 (0.62–0.93)	0.01	0.35
≥75	0.85 (0.75–0.98)	0.03	
Hypertension			
Yes	0.80 (0.71–0.90)	<0.01	0.04
No	1.32 (0.82–2.13)	0.25	
Diabetes mellitus			
Yes	0.81 (0.70–0.93)	<0.01	0.66
No	0.85 (0.71–1.02)	0.08	
Dyslipidemia			
Yes	0.86 (0.74–1.00)	0.05	0.41
No	0.781 (0.66–0.93)	0.01	
Chronic kidney disease			
Yes	0.82 (0.69–0.96)	0.02	0.80
No	0.84 (0.72–0.98)	0.03	
ACEI/ARB use			
Yes	0.81 (0.70–0.94)	<0.01	0.77
No	0.84 (0.71–1.01)	0.06	
Antiplatelet agent use ^b^			
Yes	0.80 (0.70–0.90)	<0.01	0.19
No	0.96 (0.74–1.24)	0.77	
β blocker use			
Yes	0.82 (0.68–0.98)	0.03	0.85
No	0.83 (0.72–0.96)	0.01	
Statin use			
Yes	0.83 (0.67–1.04)	0.11	0.93
No	0.82 (0.72–0.94)	<0.01	
History of influenza vaccination administration			
Yes	0.77 (0.67–0.88)	<0.01	0.85
No	0.79 (0.63–0.99)	0.04	

CI, confidence interval; ACEI, angiotensin-converting-enzyme inhibitor; ARB, angiotensin II receptor blocker.

^a^ Versus matched counterparts.

^b^ Including aspirin, clopidogrel, and ticlopidine.

## Discussion

This population-based, retrospective study demonstrated significant reductions in all-cause mortality, recurrent MI or cardiovascular death, and hospitalization for heart failure among elderly patients with previous MI who received the influenza vaccine compared with their unvaccinated counterparts. On the contrary, the influenza vaccination was not associated with hospitalization for upper gastrointestinal bleeding.

Many authors have suggested that influenza infection plays a role in precipitating CVD [5–7, 12, 27]. The incidences of influenza and CVD peak in winter [5, 27]. A large self-controlled case-series study of more than 20,000 patients in the UK showed that the incidence ratio of MI was highest during the first 3 days after acute respiratory infection, with the risk declining gradually during subsequent weeks [7]. Influenza might act as an acute inflammatory and procoagulant stimulus, transiently altering endothelial function, which might lead to destabilization of vulnerable atherosclerotic plaques and, thus, coronary artery occlusion [6, 8]. A growing body of evidence also associates influenza vaccination with a reduction in the risk of CVD among patients with previous CVD [9–13]. The association between influenza vaccination and reduced CVD is probably related to the prevention of influenza infection and consequent triggering of CVD by the mechanism mentioned above. An additional postulated mechanism is that vaccine-induced antibodies cross-react with the human bradykinin receptor, leading to increased levels of nitric oxide, which subsequently increase blood flow, vasodilation, and possibly angiogenesis [28].

Patients with CVD often suffer from recurrent episodes and have higher mortality rates [2, 3]. The association between influenza and CVD is also strongest in elderly persons [27], and the correlation coefficients increase with age [5]. Therefore, elderly people with previous CVD are at greater risk of recurrent CVD related to influenza infection and may benefit more from vaccination. In terms of primary prevention of CVD, influenza vaccination of elderly persons has been shown to be associated with substantial reductions in the risk of hospitalization for ischemic heart disease and congestive heart failure in previous studies [29, 30]. Coincident with previous findings [9, 11], our results further support the beneficial role of influenza vaccination in the secondary prevention of CVD and reduction of all-cause death in the elderly population.

Although propensity score matching may reduce healthy user bias by balancing numerous crucial individual characteristics extracted from the NHIRD, we could not control for unobserved confounders, such as health behaviors (e.g., smoking and obesity) and beliefs about and attitudes toward vaccination. The healthy vaccine effect could be mitigated only by a randomized design. Currently, two clinical trials are ongoing to determine the protective effects of influenza vaccination in patients with CVD [31, 32]. The Influenza vaccination After Myocardial Infarction (IAMI) trial [31] is a double-blind, prospective, randomized clinical trial examining whether in-hospital influenza vaccination after acute coronary syndrome protects against future CVD. By only enrolling patients not intending to be vaccinated in the current influenza season, the IAMI trial increases vaccination coverage in the target population. The randomized, double-blind, clinical Influenza Vaccine to Effectively Stop Cardio Thoracic Events and Decompensated heart failure (INVESTED) trial [32] is being conducted to compare the cardioprotective effectiveness of a high-dose trivalent influenza vaccine with that of standard-dose quadrivalent influenza vaccine in patients with high cardiovascular risk. The INVESTED trial is employing an active control, rather than placebo, and leaves no participant unvaccinated. The results of both trials, which are avoiding the potential ethical dilemma associated with vaccination research using different strategies, will be of great interest.

This study has several limitations. First, some may argue that misclassification of vaccination was possible, e.g., that some patients received influenza vaccination at their own expense, which would not be recorded in the claims data. However, we believe that this number of patients would be small because government-funded influenza vaccination has been provided to patients aged ≥ 65 years with CVD since 1998, and the out-of-pocket expenditure has been considered to be a barrier to influenza vaccination [33]. Second, the accuracy of diagnoses in the Geriatric Dataset may be of concern. However, several studies have validated the accuracy of diagnoses of diabetes [34], cerebrovascular disease [35], and acute MI [36] in the NHIRD, with positive predictive values of about 75%–90%. As the Geriatric Dataset is derived from the original NHIRD, we believe that the quality of its data is equal to that of the original NHIRD.

## Conclusion

Our study demonstrated that influenza vaccination was associated with reduced rates of secondary cardiovascular events, including all-cause mortality, recurrent MI or cardiovascular death, and hospitalization due to heart failure, in a population aged ≥ 65 years with MI.

## Supporting information

S1 Table Incidence and risk of outcomes among elderly participants with histories of myocardial infarction during the influenza season. (DOC)

S2 Table Incidence and risk of outcomes among elderly participants with histories of myocardial infarction during the year after vaccination. (DOC)
