# Supplementary material for: Influenza vaccination and secondary prevention of cardiovascular disease among Taiwanese elders—A propensity score-matched follow-up study
Source: PLoS One. 2019 Jul 1;14(7):e0219172. doi: 10.1371/journal.pone.0219172 (PMC6602195; doi:10.1371/journal.pone.0219172)
Supplement: S1 Table — (DOC) [file pone.0219172.s001.doc]

Table S1

|  | Vaccinated cohort | | | Unvaccinated cohort  (reference) | | | Crude | | Competing risk | |
| --- | --- | --- | --- | --- | --- | --- | --- | --- | --- | --- |
| Outcome | No. of events | Person-years | Incidence rate^*^ | No. of events | Person-years | Incidence rate^*^ | Hazard ratio  (95% CI) | p *-*value | Hazard Ratio  (95% CI) | p*-*value |
| All-cause death | 206 | 1,588 | 12.98 | 293 | 1,551 | 18.90 | 0.68 (0.57–0.82) | <0.001 |  |  |
| Myocardial infarction or cardiovascular death | 196 | 1,569 | 12.49 | 240 | 1,528 | 15.71 | 0.80 (0.66–0.96) | 0.018 | 0.80 (0.66–0.97) | 0.021 |
| Hospitalization for heart failure | 306 | 1,519 | 20.15 | 383 | 1,469 | 26.07 | 0.78 (0.67–0.90) | 0.001 | 0.78 (0.67–0.91) | 0.002 |
| Hospitalization for upper gastrointestinal bleeding | 30 | 1,581 | 1.90 | 33 | 1,545 | 2.14 | 0.89 (0.54–1.46) | 0.649 | 0.90 (0.55–1.48) | 0.678 |

CI, confidence interval.

*per 10^2^ person-years.
