# Supplementary material for: Influenza vaccination and secondary prevention of cardiovascular disease among Taiwanese elders—A propensity score-matched follow-up study
Source: PLoS One. 2019 Jul 1;14(7):e0219172. doi: 10.1371/journal.pone.0219172 (PMC6602195; doi:10.1371/journal.pone.0219172)
Supplement: S2 Table — (DOC) [file pone.0219172.s002.doc]

Table S2.

|  | Vaccinated cohort | | | Unvaccinated cohort  (reference) | | | Crude | | Competing risk | | |
| --- | --- | --- | --- | --- | --- | --- | --- | --- | --- | --- | --- |
| Outcome | No. of events | Person-years | Incidence rate^*^ | No. of events | Person-years | Incidence rate^*^ | Hazard ratio  (95% CI) | p *-*value | Hazard Ratio  (95% CI) | p*-*value | |
| All-cause death | 403 | 3,087 | 13.05 | 486 | 3,053 | 15.92 | 0.83 (0.72–0.94) | <0.01 |  |  | |
| Myocardial infarction or cardiovascular death | 338 | 3,031 | 11.15 | 400 | 2,990 | 13.38 | 0.84 (0.73–0.97) | 0.02 | 0.84 (0.73–0.98) | 0.02 | |
| Hospitalization for heart failure | 473 | 2,905 | 16.28 | 567 | 2,836 | 19.99 | 0.81 (0.72–0.92) | <0.01 | 0.83 (0.73–0.93) | <0.01 | |
| Hospitalization for upper gastrointestinal bleeding | 56 | 3,066 | 1.83 | 58 | 3,033 | 1.91 | 0.98(0.68–1.41) | 0.904 | 0.99 (0.68–1.43) | | 0.96 |

CI, confidence interval.

*per 10^2^ person-years.
